# Effectiveness and tolerability of intravenous pentamidine for *Pneumocystis carinii* pneumonia prophylaxis in adult hematopoietic stem cell transplant patients: a retrospective study

**DOI:** 10.1186/s12879-020-05127-y

**Published:** 2020-06-05

**Authors:** Wedad B. Awad, Alaa Asaad, Nardin Al-Yasein, Rula Najjar

**Affiliations:** Department of Pharmacy, King Hussein Cancer Center, P.O. Box 1269, Al-Jubeiha, Amman, 11941 Jordan

**Keywords:** Pentamidine, Prophylaxis, PCP, *Pneumocystis* pneumonia, *Pneumocystis jirovecii*, Hematopoietic stem cell transplantation

## Abstract

**Background:**

*Pneumocystis carinii* pneumonia (PCP) prophylaxis is recommended after hematopoietic stem cell transplantation (HSCT). In patients who are unable to take first-line prophylaxis, trimethoprim/sulfamethoxazole, aerosolized pentamidine is recommended. This drug may not, however, be available at all institutions, and its administration requires special techniques. Therefore, intravenous pentamidine (IVP) has been used in adult patients as an alternative, despite limited data. We evaluated the effectiveness and tolerability of IVP for PCP prophylaxis in adult patients who had undergone HSCT.

**Methods:**

A single-center retrospective study was conducted of adult patients who had undergone allogenic or autologous HSCT between January 2014 and September 2018 and had received at least three doses of IVP for PCP prophylaxis. The IVP dose was 4 mg/kg administered monthly. Data on PCP infection and adverse reactions were collected from both patients’ electronic medical records and the pharmacy adverse drug reactions documentation system. Patients were followed from the start of IVP up to 6 months after discontinuation of therapy. A confirmed PCP infection was defined as radiographic evidence of PCP and positive staining of a respiratory specimen. Descriptive statistics were used to analyze the study outcomes**.**

**Results:**

During the study period, 187 patients were included. The median age was 36.4 years (range, 18–64), 58% were male, and 122 (65%) had received allogeneic HSCT while the remainder autologous HSCT. The median number of IVP doses administered per patient was 5 (range, 3–29). During the study period, none of the patients had evidence of confirmed PCP infection. However; there were two cases with high clinical suspicion of PCP infection (i.e. required anti-pneumocystis therapy) and one reported case of central nervous system toxoplasmosis while receiving IVP for PCP prophylaxis. Only one case of nausea associated with IVP administration was reported.

**Conclusions:**

In a cohort of adult patients with HSCT who received IVP for PCP prophylaxis, there was no evidence of confirmed PCP infection, and the treatment appeared to be well tolerated. Prospective studies should be conducted to confirm the efficacy and tolerability of IVP.

## Background

*Pneumocystis jirovecii* (formerly *carinii*) pneumonia (PCP), is a serious fungal opportunistic infection that affects immunocompromised patients. The mortality rate associated with PCP infections in non-HIV high-risk patients is 30–60% [1, 2]. The expected risk of PCP after allogeneic hematopoietic stem cell transplantation (HSCT) is 5–15% in the absence of prophylaxis [3, 4], with a mortality rate of up to 89% in patients who develop PCP within 6 months of allogeneic HSCT and reaches up to 40% after 6 months from allogenic HSCT [3, 5].

Advances in prophylactic and treatment modalities have improved the outcome of PCP infection over the years. The guidelines of the American Society for Blood and Marrow Transplant, the Centers for Disease Control and Prevention and the European Conference on Infections in Leukemia recommend routine PCP prophylaxis in immunocompromised patients, including patients after HSCT [6–8].

The risk of infection in patients who undergo HSCT is determined primarily by the type of transplant (autologous, allogeneic), the time since transplantation, the presence or absence of graft-versus-host disease (GVHD), donor–host histocompatibility, disease status, graft type, graft content, conditioning intensity and neutrophil engraftment [6]. PCP prophylaxis is recommended for at least the initial 6 months after allogeneic HSCT [9], in patients with active GVHD who require treatment and in patients with hematological relapse or on corticosteroid use [6]. In autologous HSCT recipients, PCP prophylaxis is typically given for 3–6 months after transplantation and longer for those receiving immunosuppressive drugs [6].

Trimethoprim/sulfamethoxazole (TMP/SMX) is first-line prophylaxis against PCP after HSCT [10, 11]; however, intolerance to TMP/SMX in HSCT recipients has been reported to be as high as 55%, requiring discontinuation of the drug due to myelosuppression, rash or allergy [12]. Dapsone and aerosolized pentamidine are considered second-line therapy; they do not cause myelosuppression and are considered effective alternatives as prophylaxis against PCP infections in HSCT recipients. Dapsone may, however, cause hemolytic anemia, and aerosolized pentamidine may not be available at all institutions and requires special administration techniques and staff training that is not available at many centers, such as our institution. In addition, the cost of administration of aerosolized pentamidine is higher than that of oral therapies, and it may cause bronchospasm or dyspnea and has been associated with atypical manifestations of infection, such as atypical pneumonia and extra-pulmonary disease [10, 11].

Intravenous pentamidine (IVP) is approved by the United States Food and Drug Administration in pediatrics for both treatment and prophylaxis of PCP, while the European Medicines Agency approves it only for treatment of PCP. In adults, IVP is approved by both agencies only for the treatment of PCP, and limited available data are available to support its use in prophylaxis. It has no myelosuppressive effect and few of the adverse effects reported with inhaled pentamidine [13–15]. Given these data, we evaluated the effectiveness and tolerability of monthly IVP for prophylaxis in adult HSCT recipients.

## Methods

A retrospective study was conducted at King Hussein Cancer Center (KHCC), an internationally accredited comprehensive cancer teaching hospital in Amman, Jordan, that provides care for both adult and pediatric patients. The center accommodates a specialized adult bone marrow transplant unit, where an average of 100 transplants are performed per year.

The study included adult patients (≥ 18 years) who underwent allogeneic or autologous HSCT between January 2014 and September 2018 and received at least three consecutive doses of IVP for prophylaxis between the day of transplantation and 1 year post transplantation. Patients were excluded if they were pregnant.

According to our hospital’s protocol, all HSCT patients receive PCP prophylaxis post engraftment. TMP-SMX is standardly used as first line drug of choice. In case of poor hematopoietic engraftment or inability to tolerate oral medications, patients are started then on IVP or dapsone. In addition, patients who develop intolerance or myelosuppression with TMP-SMX use, they are switched to IVP. Duration of prophylaxis is 6 months for autologous recipients and at least 1 year for allogenic recipients. IVP for PCP prophylaxis is given as a monthly dose of 4 mg/kg of body weight infused over a minimum of 1 h, with ranitidine, hyoscine and/or metoclopramide given intravenously as pre-medication. The specific regimen for pre-medication is based on clinical judgement.

Patient data was obtained from the bone marrow transplant unit database and electronic patient profiles. The list of HSCT patients who received IVP was extracted from the pharmacy billing system for the study period, filtered according to the number of IVP doses received and then matched with the adult bone marrow transplant unit database. The data were then entered into a de-identified, secured central database.

PCP infection work-up is performed for any patient with respiratory symptoms after consultation with the adult infectious disease specialist. The workup consisted of obtaining respiratory specimen through bronchoalveolar lavage for those with suspected PCP infection based on the chest computed tomography scan. PCP infection was identified by reviews of patients’ electronic medical records for any clinical, radiographic or microbiological evidence, and positive (dye-based) or fluorescent antibody staining of respiratory specimens. Any PCP infection identified after the start of IVP prophylaxis was considered as failure of the regimen. Patients were followed-up for at least 6 months after discontinuation of IVP. Furthermore, Patients’ electronic medical profiles were evaluated for any evidence of toxoplasmosis infection while on IVP for PCP prophylaxis.

The incidence of adverse drug reactions after administration of IVP was obtained from both patients’ electronic profile reviewed for 1 week after each administered dose of IVP and the pharmacy online documentation system for adverse drug reactions.

Descriptive statistics were used to analyze all background and patient demographic data as well as the effectiveness and tolerability outcomes. Categorical data were reported as absolute and relative frequencies, while continuous data were reported as means and standard deviations or medians and ranges.

## Results

**Fig. 1:**
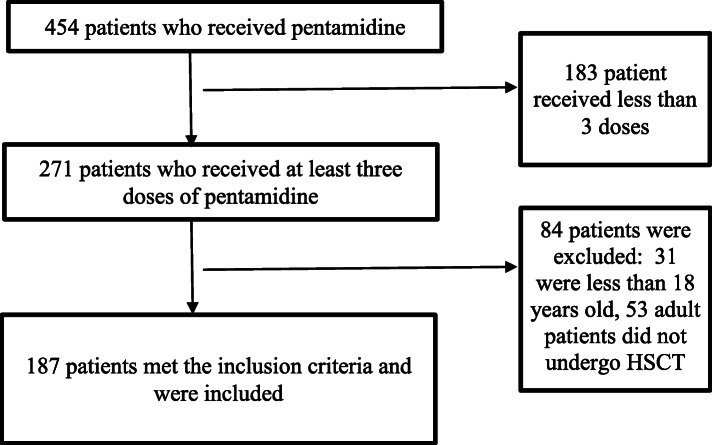
Flow diagram of included patients

A total of 454 patient who received pentamidine during the study period were identified. Among those, 183 patients received less than 3 doses of pentamidine, and therefore were excluded from the cohort. For those patients, IVP was switched to oral TMP-SMX due to improvement in their hematological counts and ability to continue on first line therapy as per our institution’s standard protocol. None of them were stopped due to development of PCP or intolerance to pentamidine. Of the remaining 271 transplant recipients, 187 met the inclusion criteria. The remaining 84 patients were excluded as they either did not undergo HSCT and/or they are less than 18 years of age (Fig. 1). All 187 patients received a dose of 4 mg/kg, and 182 received one or more of the pre-medications. Patient baseline characteristics included demographics, primary diagnosis, conditioning regimen intensity, transplant stem cell source, HLA matching for patients undergoing allogeneic transplant, incidence of acute and chronic GVHD, and relevant co-morbidity (Table 1).

**Table 1:** Baseline characteristics of patients

Baseline characteristic	Value (*N* = 187)
Age (years), median (range)	36.4 (18–64)
Gender, N (%)	
Male	108 (57.7%)
Primary diagnosis, N (%)	
Leukaemia	77 (41.2%)
Acute lymphoblastic leukaemia	19
Acute myeloid leukaemia	39
Chronic myeloid leukaemia	7
Myelodysplastic syndrome	12
Lymphoma	66 (35.3%)
Non-Hodgkin lymphoma	24
Hodgkin lymphoma	42
Other malignancies	16 (8.5%)
Multiple myeloma	15
Germ-cell tumour	1
Non-malignancies	28 (14.9%)
Aplastic anaemia	14
Fanconi anaemia	1
Myelofibrosis	2
Red-cell aplasia	1
Sickle-cell anaemia	1
Thalassaemia	9
Type of transplant	
Autologous	65 (34.8%)
Allogeneic	122 (65.2%)
Related donor	15
Unrelated donor	105
Haplo-identical transplant	2
Stem cell source, N (%)	
Peripheral stem cells	166 (88.8%)
Bone marrow cells	19 (10.1%)
Bone marrow and peripheral stem cells	1 (0.5%)
Umbilical cord bank	1 (0.5%)
Conditioning regimen intensity, N (%)	
Myeloablative	130 (69.5%)
Reduced intensity	55 (29.4%)
Reduced toxicity	1 (0.5%)
None	1 (0.5%)
Baseline comorbidity, N (%)	
Cardiac disease	40 (21.4%)
Endocrine disease	25 (13.3%)
Kidney disease	10 (5.3%)
Liver disease	6 (3.2%)
Other	43 (22.9%)
GVHD, N (%)	
Acute	16 (8.6%)
Chronic	31 (16.6%)
Both acute and chronic	38 (20.3%)
None	102 (54.5%)

Of the 187 patients, 122 (65.2%) had undergone allogeneic bone marrow transplantation and 65 patients (34.8%) autologous bone marrow transplantation. The two primary indications for HSCT were leukemia and lymphoma, representing 41.2 and 35.3% of the patients; respectively. HSCT was performed in 14.9% of the patients for a non-malignant condition.

The majority of patients (82.4%) received up to eight monthly doses of IVP prophylaxis; 11.2% continued the prophylaxis for 9–12 doses and 6.4% for > 1 year. The median number of doses was 5 (range, 3–29) (Table 2). Patients were started on IVP at a mean of day 31 post-transplant, with a median of 25 days (range, 1–127). During the study period, none of the patients had evidence of confirmed PCP infection. Although 32 patients were evaluated for suspected PCP; none had confirmed diagnosis and only two patients continued to receive treatment despite negative respiratory specimens due to a high clinical suspicion of PCP. Only one patient was reported to have central nervous system toxoplasmosis while receiving IVP for PCP prophylaxis.

**Table 2:** Pentamidine-related data

Variable	Value
Number of pentamidine doses, median (range)	5 (3–29)
3–5	106
6–8	48
9–12	21
> 12	12
Complete blood count at first pentamidine dose, N (%)	
Normal ANC^a^ and platelet count	80 (42.6%)
Low ANC^a^ and platelet count	96 (51.6%)
Could not be obtained	11 (5.9%)
Day of IVP initiation after transplantation, median (range)	25 (1–127)
Choice of IVP for prophylaxis, N (%)	
First choice	136 (72.7%)
Second choice	51 (27.3%)

^a^*ANC* Absolute neutrophil count

IVP was used as the first choice for PCP prophylaxis for 136 patients (72.7%); the others were started initially on TMP/SMX or dapsone per standard care and then switched to IVP due to intolerability.

Five patients received no pre-medication before IVP administration; none had a documented adverse drug reaction. Among the study population, only one patient reported nausea as an adverse event after administration of IVP despite receiving proper pre-medications.

## Discussion

This retrospective study supports the use of IVP in adult HSCT patients with no documented confirmed PCP infections. Although TMP-SMX remains the first line drug choice for prophylaxis against PCP, given its effectiveness against *Pneumocystis jirovecii* and other opportunistic infections, as well as its relatively low cost, second line agents such as IVP may be necessary in cases of intolerance, sulfa allergy and myelosuppression.

Regarding dosing of IVP, the administered IVP dose in our study was 4 mg/kg per month infused over a minimum of 1 h after pre-medication with IV ranitidine, hyoscine and/or metoclopramide. Sweiss et al. [16] reported an IVP dose of 4 mg/kg (with a maximum of 300 mg per dose) infused over a standard infusion time of 2 h and the pre-medication was ondansetron in their study. Another study, Diri R et al. [3], reported use of a standard IVP dose of 300 mg and pre-medication with diphenhydramine and ondansetron before infusion. Furthermore, our patients were started on IVP at a mean of 31 days after transplant, while in comparison with other studies [3, 16] IVP was started at any time after the end of conditioning chemotherapy or within 6 days of the scheduled allogeneic transplantation.

The incidence of PCP infection in adult HSCT patients who received IVP for prophylaxis was reported in two studies [3, 16], one prospective and the other retrospective. Both studies reported no PCP infection, as in our study. The study population in the prospective study consisted of adults who had undergone HSCT or had received only intensive chemotherapy, and the retrospective study included only patients who had undergone allogeneic HSCT patients. These findings are consistent with those reported in the literature in pediatric HSCT population [11, 13, 17, 18]. However, a concern toward an increased risk of breakthrough PCP infection in younger patients receiving IVP as PCP prophylaxis was reported in the pediatric population [11].

In term of IVP tolerability, injection site reaction, renal insufficiency, hypotension, gastrointestinal discomfort, leukopenia, azotemia, increased liver enzymes, skin rash and flushing were possible adverse events reported in the literature following IVP administration [19, 20]. We had only one documented adverse event among our patients that was nausea. Sweiss et al. [16] reported in their prospective study that nausea (8%) and hypotension (12%) as common adverse events in their patients, and less commonly nasal congestion (4%), oral numbness (4%), infusion related reaction (4%), acute kidney injury (4%) and rash (2%). Nevertheless, all reported adverse events were of grade I/II and none had reported grade III/IV adverse events. In pediatrics, DeMasi J et al. [18] reported nausea/vomiting (7.3%), grade IV reaction with anaphylaxis (rash) and hypotension (1.5%) as adverse events post IVP administration.

This study included a relatively large sample size of both allogeneic and autologous adult HSCT patients, which is larger than previously reported studies. However, our study had several limitations. One is being a retrospective study and therefore there is a potential of missing some of data especially concerning adverse drug reactions. Secondly, the adverse drug reactions were identified through the pharmacy documentation reporting system and electronic patient profiles, and thus some might have been missed due to underreporting. Another limitation to our study is related to the diagnostics used, since quantitative real time PCR was not done in our patients due to its unavailability at our center. This may be a limitation as PCR is considered to have better sensitivity in immune compromised patients, compared to the microscopic examination used in our patients [21]..

## Conclusions

Our findings support the use of IVP for PCP prophylaxis in adult HSCT patients. Prospective studies should be conducted to confirm the efficacy and tolerability of IVP.

## Data Availability

The datasets used and/or analysed during the current study are available from the corresponding author on reasonable request.

## Abbreviations

TMP/SMX: Trimethoprim/sulfamethoxazole
IVP: Intravenous pentamidine
KHCC: King Hussein Cancer Center
IV: Intravenous
HSCT: Hematopoietic stem cell transplantation
GVHD: Graft versus host disease
